# 61 Effect of Pre-Existing Hypertension on Cardiovascular Support and Mortality Among Major Burn Patients

**DOI:** 10.1093/jbcr/irad045.035

**Published:** 2023-05-15

**Authors:** Trina Stephens, Anthony Papp, Bettina Papp, Sara Sheikh-Oleslami, Donald Griesdale, Fagun Jain

**Affiliations:** University of British Columbia, Vancouver, British Columbia; University of British Columbia, Vancouver, British Columbia; University of British Columbia, Vancouver, British Columbia; University of British Columbia, Vancouver, British Columbia; University of British Columbia, Vancouver, British Columbia; University of British Columbia, Vancouver, British Columbia; University of British Columbia, Vancouver, British Columbia; University of British Columbia, Vancouver, British Columbia; University of British Columbia, Vancouver, British Columbia; University of British Columbia, Vancouver, British Columbia; University of British Columbia, Vancouver, British Columbia; University of British Columbia, Vancouver, British Columbia; University of British Columbia, Vancouver, British Columbia; University of British Columbia, Vancouver, British Columbia; University of British Columbia, Vancouver, British Columbia; University of British Columbia, Vancouver, British Columbia; University of British Columbia, Vancouver, British Columbia; University of British Columbia, Vancouver, British Columbia; University of British Columbia, Vancouver, British Columbia; University of British Columbia, Vancouver, British Columbia; University of British Columbia, Vancouver, British Columbia; University of British Columbia, Vancouver, British Columbia; University of British Columbia, Vancouver, British Columbia; University of British Columbia, Vancouver, British Columbia; University of British Columbia, Vancouver, British Columbia; University of British Columbia, Vancouver, British Columbia; University of British Columbia, Vancouver, British Columbia; University of British Columbia, Vancouver, British Columbia; University of British Columbia, Vancouver, British Columbia; University of British Columbia, Vancouver, British Columbia; University of British Columbia, Vancouver, British Columbia; University of British Columbia, Vancouver, British Columbia; University of British Columbia, Vancouver, British Columbia; University of British Columbia, Vancouver, British Columbia; University of British Columbia, Vancouver, British Columbia; University of British Columbia, Vancouver, British Columbia

## Abstract

**Introduction:**

Hypertension (HTN) is the most common comorbidity seen in patients who sustain a major burn. HTN causes increased responsiveness of the autonomic nervous system to stressful stimuli, activation of RAAS, arterial and myocardial hypertrophy, and impaired vasodilation. Changes to these pathways are also seen in response to major burns. Given this common pathophysiology, patients with pre-existing HTN may demonstrate different mortality risk after major burn than those without HTN. The objective of this study is to compare the risk of in-hospital mortality among adult patients with and without pre-existing HTN admitted to the intensive care unit (ICU) with a major burn. In addition, we sought to determine the association of pre-existing HTN with the need for vasoactive agents, fluid resuscitation and urine output in the first 48-hours of ICU admission

**Methods:**

Single-centre, historical cohort study using data from a Burn Registry, Intensive Care Database, and medical chart review. Variables of interest were demographic and injury characteristics, use of vasoactive agents, 12-, 24-, and 48-hour fluid requirements and urine output, and in-hospital mortality. We included adult (≥18 years) patients presenting with ≥20% total body surface area (TBSA) burns who were admitted to the ICU from January 2010 to August 2021. Pre-existing HTN was defined by pharmacy records showing the patient had filled a prescription for an anti-HTN medication within the 12-months prior to their burn. We compared variables of interest by HTN exposure and conducted univariate analysis to assess mortality risk

**Results:**

199 patients met inclusion criteria. Of these, 145 records were complete. The mean (standard deviation, SD) age was 47 (7) years and TBSA was 37 (16) percent. Most patients were males (83.5%) who sustained a flame burn (87.6%) without an inhalation injury (28.5%). Pharmacy records revealed that 28 patients (19.3%) had pre-existing HTN. Mean (SD) 24-hour fluid requirements were 7.1 (5.6) L and 5.2 (3.2) L for HTN and non-HTN patients, respectively (p=0.17). Mean (SD) 24-hour urine output was 1024 (747) mL and 1314 (780) mL for HTN and non-HTN patients, respectively (p=0.12). Sixty-one percent of HTN patients required vasoactive agents in the first 24-hours compared to 46.2% of non-HTN patients (p=0.18). Pre-existing HTN was associated with a 5-fold increase in in-hospital mortality (OR 5.5, CI 2.2-14.0)

**Conclusions:**

Preliminary results suggest differences in fluid requirements, urine output and mortality risk between HTN and non-HTN patients with major burns. An external registry has been queried to identify pharmacy records for the 54 patients with missing data

**Applicability of Research to Practice:**

Identifying differences in mortality risk and need for cardiovascular support among burn patients with HTN changes our approach to counselling and caring for these patients

